# Task-specific auditory distraction in serial recall and mental arithmetic

**DOI:** 10.3758/s13421-022-01363-6

**Published:** 2022-10-14

**Authors:** Florian Kattner, Sarah Hanl, Linda Paul, Wolfgang Ellermeier

**Affiliations:** 1 Health and Medical University, Olympischer Weg 1, 14471 Potsdam, Germany; 2 Technical University of Darmstadt, Darmstadt, Germany

**Keywords:** Irrelevant sound effect, Auditory distraction, Serial recall, Mental arithmetic, Changing-state effect, Deviation effect

## Abstract

Previous studies suggest that task-irrelevant changing-state sound interferes specifically with the processing of serial order information in the focal task (e.g., serial recall from short-term memory), whereas a deviant sound in the auditory background is supposed to divert central attention, thus producing distraction in various types of cognitive tasks. Much of the evidence for this distinction rests on the observed dissociations in auditory distraction between serial and non-serial short-term memory tasks. In this study, both the changing-state effect and the deviation effect were contrasted between serial digit recall and mental arithmetic tasks. In three experiments (two conducted online), changing-state sound was found to disrupt serial recall, but it did not lead to a general decrement in performance in different mental arithmetic tasks. In contrast, a deviant voice in the stream of irrelevant speech sounds did not cause reliable distraction in serial recall and simple addition/subtraction tasks, but it did disrupt a more demanding mental arithmetic task. Specifically, the evaluation of math equations (multiplication and addition/subtraction), which was combined with a pair-associate memory task to increase the task demand, was found to be susceptible to auditory distraction in participants who did not serially rehearse the pair-associates. Together, the results support the assumption that the interference produced by changing-state sound is highly specific to tasks that require serial-order processing, whereas auditory deviants may cause attentional capture primarily in highly demanding cognitive tasks (e.g., mental arithmetic) that cannot be solved through serial rehearsal.

## Introduction

Task-irrelevant non-stationary sounds such as background speech, random tone sequences, or music are known to disrupt verbal short-term memory (the “irrelevant sound effect”, e.g., Colle & Welsh, 1976; Jones & Macken, 1993; Salamé & Baddeley, 1982, 1989). In a typical irrelevant sound paradigm, participants are asked to memorize sequences of verbal items typically presented visually on a screen (e.g., digits, letters, or words) for a short period while different types of to-be-ignored sound is presented. In many early studies, the presence of irrelevant speech was found to produce considerable impairment of serial recall performance, compared to a quiet or noisy background, regardless of whether the speech consisted of words or non-words, whether it was presented in a familiar or unfamiliar language, or whether it was played forward or backward (e.g., Ellermeier & Zimmer, 1997; Jones et al., 1990; LeCompte et al., 1997; Salamé & Baddeley, 1982). While the to-be-remembered items are usually presented visually, similar disruptive effects of irrelevant sound were demonstrated also for serial recall of auditory verbal items (e.g., Campbell et al., 2002; Jones et al., 2004; Kattner & Ellermeier, 2020; Nicholls & Jones, 2002). In addition, distraction of serial recall can be demonstrated also with various types of non-speech sounds such as random tone sequences (e.g., Jones et al., 1999; Jones & Macken, 1993; LeCompte et al., 1997), instrumental music (e.g., Kattner & Meinhardt, 2020; Nittono, 1997; Schlittmeier et al., 2008; Williamson et al., 2010), or office noise (e.g., Bell & Buchner, 2007; Schlittmeier & Hellbrück, 2009). It has been argued that the degree of distraction may depend on certain psychoacoustic properties of the irrelevant sound, such as the presence of pitch changes, fluctuation strength, or speech-specific features (for a review, see Ellermeier & Zimmer, 2014).

While the disruptive effect of irrelevant speech on verbal short-term memory has been explained originally with phonological interference-by-content (i.e., due to obligatory processing of speech in the phonological loop system; Baddeley & Hitch, 1974), it has been argued later that the presence of “changing-state” information in irrelevant sound produces task-specific interference with serial-order processing (the “interference-by-process account”; Jones et al., 1992, 2004, Jones et al., 1996). In particular, the disruption of serial short-term memory may be due to the automatic perceptual tracking of acoustical changes in the background sound, which gives rise to the formation of discrete auditory objects during auditory scene analysis (see Bregman, 1990; Handel, 1989). During this perceptual organization process, sequences of auditory objects are grouped together as streams using order cues (“pointers”), which can be used also for the deliberate rehearsal and retrieval of serial order information in short-term memory. However, any irrelevant stream of ordered auditory objects may then interfere with retention of the serial order in the to-be-remembered stream of items. In line with this account, it was found that temporally varying (changing-state) sound produces more disruption in serial recall than repeated sounds, regardless of its phonological content (Jones & Macken, 1993). Moreover, the disruption of serial recall was found to increase both with the magnitude (e.g., pitch distance between successive sounds; Jones et al., 1999) and number of acoustical changes in irrelevant sound (e.g., the “token set size effect”; Tremblay & Jones, 1998), whereas repeated tones or syllables (i.e., “steady-state sound”) do not produce disruption compared to silence. However, more recently it has been reported that steady-state sound may also produce a small disruptive effect compared to silence, which can be detected with sufficient statistical power (Bell, Röer, et al., 2019a). Observations such as the steady-state effect cannot be explained with an account that assumes interference-by-process to be the only mechanism of auditory distraction in serial recall. Hence, auditory distraction must be explained either with a different mechanism (e.g., automatic attentional capture; Bell et al., 2012; Cowan, 1999) or an account that assumes two (or more) distinct mechanisms of distraction (e.g., the duplex-mechanism account; Hughes et al., 2005).

According to the *unitary attentional account* of auditory distraction, an irrelevant sound is expected to elicit an orienting response diverting attentional resources from the focal task to the irrelevant sound. The degree of attentional capture should depend on the mismatch between the distractor sound and the neural, predictive model based on previous stimulation (e.g., Bell et al., 2010; Escera et al., 1998). As repetitions of the same stimuli help the formation of an accurate predictive model, which leads to an adaptation of the orienting response (i.e., reduced prediction errors), the model can explain why steady-state sound produces less distraction than sequences consisting of two or more discrete sounds (Bell, Röer, et al., 2019a, 2019b). Also in line with the attentional account, it has been found that the presentation of the same sequences of irrelevant speech leads to habituation of the disruptive effect on serial recall (as compared to silence; Bell et al., 2012; Röer et al., 2014), and that foreknowledge of the distractor information may reduce the additional disruption (beyond the changing-state effect), which is produced by semantic or syntactic/grammatical properties of complex irrelevant speech (i.e., due to stimulus-specific attentional capture; Hughes & Marsh, 2020; Röer, Bell, & Buchner, 2015a). However, what is more difficult to explain with an attentional predictability account is the observation that complex speech (meaningful sentences) produce more disruption than changing-state sound of low predictability (e.g., random letters; Hughes & Marsh, 2020).

According to the alternative *duplex-mechanism account*, auditory distraction can be the result of two functionally distinct mechanisms: interference-by-process and attentional capture (Hughes, 2014; Hughes et al., 2005, 2007; Hughes & Marsh, 2017; Jones & Macken, 2018). That is, changing-state sound is expected to produce specific interference with serial-order processing (i.e., general-purpose motor-planning processes that are recruited for the retention of serial information; Hughes & Marsh, 2017; Jones & Macken, 2018), but irrelevant sound may also divert attention from the focal task either due to a mismatch with the neural model based on previous stimulation (e.g., a temporal irregularity or an unexpected change in voice; Hughes et al., 2005, 2007), or due to a particular content in irrelevant speech (e.g., a taboo word; Röer et al., 2017). In line with the specific interference-by-process assumption (Jones et al., 1996; Jones et al., 2004), the disruptive effect of changing-state sound, compared to steady-state sound, was found to be specific to serial short-term memory tasks, whereas tasks that do not require serial-order processing seem to be immune to a changing-state effect. A prototypical example of a non-serial task is the “missing-item task” (Buschke, 1963), in which one item from a predetermined list (e.g., the numbers from 0 to 9, or the days of the week) is missing in the presented sequence, and participants are asked to recall only the missing item. Several studies found that performance in this task was unaffected by the presence of changing-state sound (Beaman & Jones, 1997; Hughes et al., 2007; Jones & Macken, 1993; Perham et al., 2007). However, other researchers demonstrated disruptive effects of irrelevant speech both in free recall and missing item tasks, but they did not observe a difference between the disruptions by changing-state and steady-state speech on performance in the missing item task (LeCompte, 1994, 1996). In addition, auditory distraction has been demonstrated also in other verbal tasks such as reading, which may not necessarily involve serial-order processing (e.g., proofreading and reading comprehension tasks; Halin et al., 2014; Jones et al., 1990; Vasilev et al., 2019). While these findings suggest that the disruptive effect of irrelevant changing-state sound or speech may not be restricted to serial recall, it has been debated to what extent participants may still have used serial rehearsal as a retention strategy in free recall or missing-item tasks (Jones, 1995). Indeed, the disruptive effect in the missing item task seems to depend on the particular memorization strategy used. For instance, performance in this task was found to be unaffected by irrelevant speech if the complete stimulus set (e.g., religious buildings) had been learned previously in alphabetical order, thus providing participants with a long-term representation of the order of items in the set, which allows for a non-serial “checking-off” strategy. In contrast, if the stimulus set had been learned in random order, recall of the missing item was clearly impaired by the presence of irrelevant speech, presumably because participants had to resort the order of the to-be-encoded items for recall of the missing item (i.e., they engaged in serial-order processing; Beaman & Jones, 1997). More recent evidence also suggests that that the disruptive effect of changing-state sound in the missing-item task crucially depends on whether or not participants reported to have used a serial rehearsal (or grouping) strategy (Hughes & Marsh, 2020).

In addition to the presumably task-specific and largely automatic changing-state effect, attentional capture may lead to a more general disruption of cognitive performance, which should not be restricted to tasks that require serial-order processing. Specifically, in contrast to the unitary attentional account, the duplex-mechanism account explains the disruptive effect of a single unpredicted or salient auditory item (a deviant) with a different task-unspecific mechanism than task-specific interference produced by changing-state sound. Consistent with this prediction, auditory deviation effects were observed in various cognitive tasks including perceptual classification tasks (e.g., duration judgments; Leiva et al., 2015; Li et al., 2013; or even/odd digit categorizations; Parmentier et al., 2008; Schröger & Wolff, 1998), the missing-item task (Hughes et al., 2007), and different forms of verbal and spatial serial recall (e.g., Bell, Mieth, et al., 2019; Hughes & Marsh, 2019; Kattner & Ellermeier, 2018; Marois et al., 2019; Vachon et al., 2017). However, the empirical evidence for the dissociation in terms of the task-specificity between the changing-state and deviation effect is based almost entirely on the comparison between serial recall and the missing-item task, whereas other (presumably less “serial”) tasks were shown to be equally susceptible to the changing-state effect (Beaman & Jones, 1998; LeCompte, 1994). Certainly, the missing-item task is an excellent choice of a non-serial short-term memory task, because it shares many of its characteristics with the serial recall task (e.g., the presented items and the sequence length). However, it has also some weaknesses such as the lower sensitivity as a memory measure (i.e., only one item needs to be recalled) and the heterogeneity of mnemonic strategies that can be used to recall the missing item (e.g., in Hughes & Marsh, 2020 Exp. 2, 40% of the participants used serial rehearsal or grouping and 60% used other strategies such as a “checking the items off as they arrived”). In order to demonstrate the generalizability of a dissociation between two forms of auditory distraction (beyond the highly artificial missing-item laboratory task), it is important to investigate auditory distraction also in other, more ecologically valid cognitive tasks that are unlikely to involve serial-order processing in short-term memory. A mental arithmetic task may be another good candidate to compare to the serial recall task because it also involves the processing of sequentially presented digits, but it can be designed in a way that eliminates the demand for serial order processing. For instance, in a task with successive additions and subtractions of numbers presented on the screen (e.g., Banbury & Berry, 1998), participants are required to mentally add/subtract new numbers to/from a previously presented or calculated single number. In such a task, only the most recent number needs to held and updated in working memory (e.g., through subvocal rehearsal of the single number) while there is no need to process or rehearse the order in which the numbers were presented (i.e., the previous number becomes irrelevant as soon as the new result has been calculated). In other words, the task is not expected to involve much serial-order processing, and serial rehearsal and grouping strategies (i.e., the main serial-processing strategies; Hughes & Marsh, 2020) are unlikely to be of any benefit in this task (memorizing the order of digits may be a counterproductive waste of cognitive resources). Hence, if the changing-state effect was specific to serial-order processing (in line with the duplex-mechanism account), it should not be observed in such a mental arithmetic task, whereas the task is likely to be susceptible the auditory deviation effect due to unspecific attentional capture. In contrast, the unitary attentional account predicts that changing state sound (due to its low predictability) should divert more attention than steady-state sound from the cognitive processes that are involved in mental arithmetic as well.

There is some previous evidence of irrelevant speech and other types of sound to disrupt performance in mental arithmetic tasks (Banbury & Berry, 1998; Perham et al., 2013, 2016; Perham & Macpherson, 2012). Banbury and Berry (1998), for instance, demonstrated that the presence of irrelevant speech and office noise (as compared to silence) reduces the accuracy in a task that requires alternating additions and subtractions of single digits (e.g., 8+7–2+5–1+8–4+7–3+5–8+4–1+6–7=?). While this task may involve some short-duration serial rehearsal (e.g., subvocal grouping the last interim result with the new digit), longer serial rehearsal does not seem to be an effective strategy to add and subtract digits. Likewise, it has been shown that performance on the same mental arithmetic task is disrupted by the presence of changing-state speech utterances and tones, compared to silence (and both effects were more pronounced on the left ear; Hadlington et al., 2006). However, due to the lack of auditory deviants and a steady-state control condition in these studies, it is still unclear whether these disruptive effects on mental arithmetic performance are due to (a) interference with serial-order processing (it has been debated whether both mental arithmetic and serial recall may involve serial-order processing in the right hemisphere; see Hadlington et al., 2006; p. 163) or (b) the diversion of attentional resources from the focal task.

According to the unitary attentional account (Bell et al., 2012; Cowan, 1999), both changing-state sound and auditory deviants are expected to be due to the same mechanism (unspecific attentional capture) and should thus disrupt performance not only in serial recall, but also in mental arithmetic tasks. In contrast, the duplex-mechanism account (Hughes, 2014; Hughes et al., 2005) predicts that changing-state sound should only affect serial recall (due to interference-by-process), but not the mental arithmetic task, whereas auditory deviants are expected to disrupt performance in both task, due to a general diversion of attention form the focal task.

In the present study, the disruptive effects of changing-state sound and auditory deviants were contrasted between serial recall and different types of mental arithmetic tasks, for which serial rehearsal is not likely to be the predominant strategy. Specifically, the first objective was to test whether the disruptive effect of changing-state sound (compared to steady-state sound) is restricted to serial recall and does not affect mental arithmetic performance. The second objective was to test whether an auditory deviant in steady-state or changing-state sequence of irrelevant sound (i.e., a change in voice) disrupts performance in both tasks through unspecific attentional capture. The mental arithmetic tasks were designed to have procedural properties as similar as possible to the serial recall task, including the presentation of visual digits at the same rate (in contrast to Banbury & Berry, 1998, both the digits and addition/subtraction operators were presented for a fixed duration – not self-paced by the participant).

## Experiment 1

### Method

#### Participants

Thirty-one participants (22 women, nine men) were recruited at Technical University of Darmstadt. Ages ranged between 18 and 35 years (*M* = 22.1; *SD* = 5.1). A sensitivity analysis of statistical power (using G*Power 3.1.9.7; Faul et al., 2007) revealed that this sample size was sufficient to demonstrate an interaction between the type of sound (e.g., changing-state vs. steady-state) and the type of task with an effect size of *f* = .30 or larger with a statistical power of 90% (α = .05; correlation *r* = .5 among repeated measures, no sphericity correction). The sample size thus appears appropriate to detect a modulation of the changing-state effect as a function of the type of task similar to previous reports of sound × task interactions (e.g., the interaction of the changing-state effect with the type of task, serial recall vs. missing item task, *f* = 0.36; see cross-experiment analysis in Hughes et al., 2007; p. 1056).

All participants reported normal hearing and normal or corrected-to-normal vision. The majority of participants (*n* = 27) were students who were compensated with partial course credit, the remaining participants agreed to take part in the experiment without financial compensation. Each participant gave written informed consent before starting the task. The ethics committee of the Technical University of Darmstadt approved the protocol of Experiment 1 on 26 November 2019 (EK 42/2019).

#### Apparatus

The experiment was conducted in a double-walled sound-attenuated listening booth (Industrial Acoustics Company, Niederkrüchten, Germany). The routines for stimulus presentation and response measurement were programmed in Matlab (MathWorks, Natick, MA, USA) utilizing the Psychophysics Toolbox 3.0 extensions (Brainard, 1997; Kleiner et al., 2007; Pelli, 1997). Visual stimuli were displayed on a 19-in. LCD monitor (Zalman Trimon 190B) inside the listening booth. Audio signals were D/A converted by an external sound card (RME multiface II; Audio AG, Haimhausen, Germany) at a sampling rate of 44.1 kHz (32 bits) and amplified (Behringer HA 800 Powerplay Pro-8; Behringer, Zhongshan, China) before being played diotically via headphones (Beyerdynamics DT-990 Pro; Beyerdynamic GmbH, Heilbronn, Germany).

#### Irrelevant sound

Speech recordings of 15 unique monosyllabic German consonant names (b, f, g, h, k, l, m, n, p, q, r, s, t, w, and x) were made with a male and a female speaker. Each recording was cut to 700 ms. Different auditory sequences of 19.6 s duration were created, each consisted of 28 consonant recordings. The steady-state sequences consisted of 28 repetitions of a single randomly drawn consonant, each spoken by the male voice. The changing-state sequences consisted of 28 consonants that were drawn randomly with replacement from the set of 15 consonants spoken by the male voice. On deviant trials, the 12th consonant in the sequence (steady- or changing-state) was replaced by a recording of the same consonant with the female voice (i.e., the deviant started 7.7 s and ended 8.5 s after the onset of the auditory sequence, corresponding approximately to the presentation of the fourth visual target item; see below). Unique auditory sequences were generated randomly for each participant.

#### Experimental design

To test for both additive and interactive effects of changing-state sound and auditory deviants, a full-factorial 2 (task: serial recall, mental arithmetic) × 2 (state of sound: steady-state, changing state) × 2 (deviant: present, absent) repeated-measures design was implemented. Each participant completed 60 trials of serial recall and 60 trials of mental arithmetic. Within each task, steady-state and changing-state sound was presented on half of the trials each. On 20% of all trials, the auditory sequence contained a voice deviant (i.e., six trials per task and sound condition).

#### Procedure

The experiment started with four practice trials containing two trials of serial recall and two mental arithmetic trials (one with steady-state and one with changing-state sound). Each participant then completed the serial recall and mental arithmetic tasks randomly intermixed across a total of 120 trials. Prior to each trial, a text message was presented for 1.5 s informing the participants whether the task was to memorize the digits in serial order (serial recall) or to mentally add and subtract the numbers (mental arithmetic).

In the serial recall task, eight digits were drawn randomly without replacement from 1 to 9 and presented in black on a grey screen at a rate of 2 s per digit. The digits were followed by a 4-s retention interval showing a blank grey screen. During both the presentation of digits and the retention interval, an irrelevant auditory sequence was presented. Participants were instructed to ignore the sound and to focus on the digits. After the retention interval, a numeric pad was shown on the screen, and participants were asked to click the digits the order they had been presented. Feedback was given for 1 s immediately after the last response, showing the number digits that were recalled in the correct serial position.

In the mental arithmetic task (similar to Banbury & Berry, 1998), participants were also presented with a sequence of eight digits at a rate of 2 s per digit, with each (except for the first digit) being accompanied by either a plus or a minus sign (e.g., “+3” or “–8”). The sign indicated whether the current digit was to be added to or subtracted from the previous result. After the last digit, there was a 4-s retention interval before participants were asked to enter the final result of the additions and subtractions. Therefore, a numeric response pad with the digits 0–9 and an additional minus sign was shown on the screen, and participants were asked to click the result and to confirm the result without the option to correct their response. Feedback was presented for 1 s after the response had been confirmed indicating whether the result was correct or not. Irrelevant sound was presented during both digit presentation and the retention interval.

#### Data analysis

Performance on the two tasks was analyzed as a function of the type of sound with classical and Bayesian repeated-measures analyses of variance (ANOVA) using JASP 0.16.2. For the Bayesian ANOVA, random slopes were included for all repeated-measures factors except the highest-order interaction (the new recommended method as suggested by van den Bergh et al., 2022). Inclusion Bayes factors (*BF*_*Incl*_) were calculated based on the Bayesian ANOVA model to provide an indication of the likelihood of the data given a model that contains the particular term of interest. Specifically, *BF*_*Incl*_ *> 1* indicates the extent to which a particular main effect or interaction term improves the model fit in comparison to models without the particular term of interest (for main effects: models that do not include an interaction with the particular term of interest; for interactions: by averaging across all models containing main effects from the interaction term). Individual alpha-adjusted post hoc comparisons were conducted in R.

### Results

**Fig. 1:**
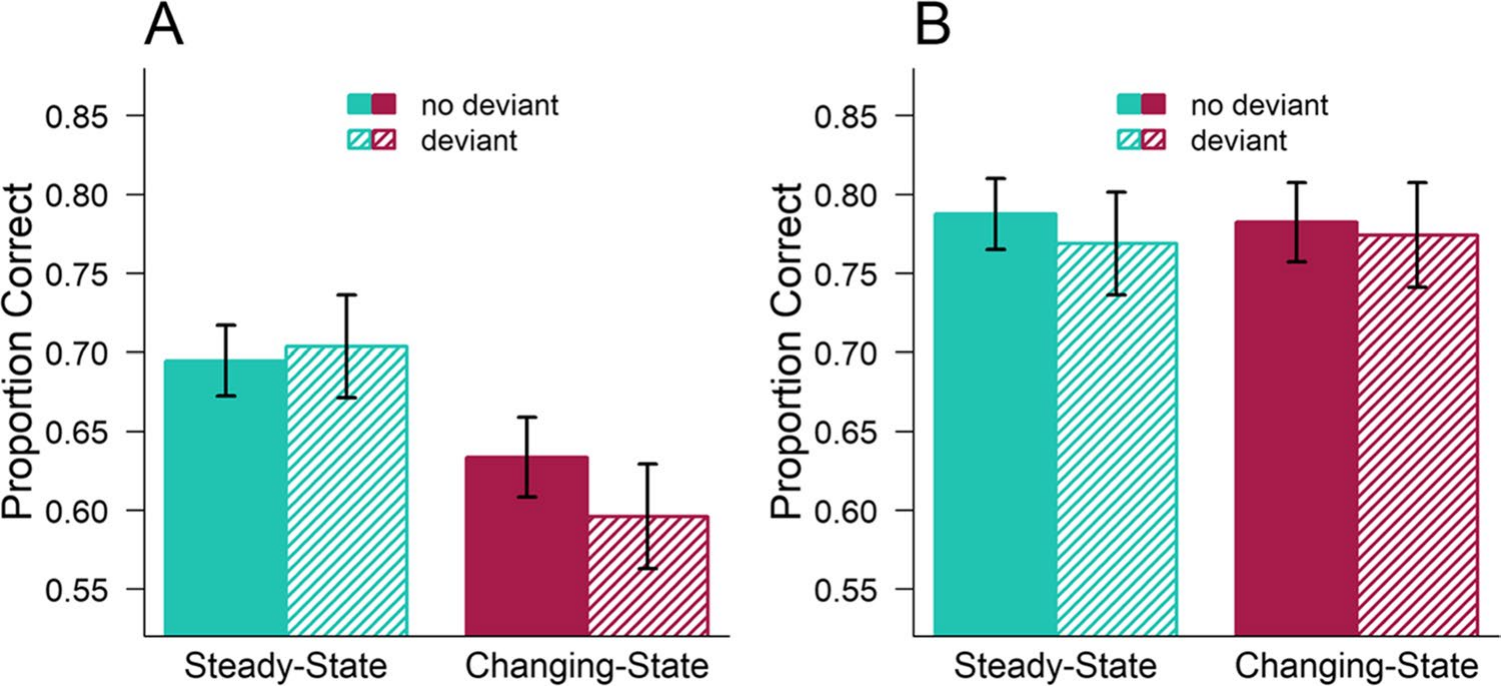
Mean accuracy in (**A**) the serial recall and (**B**) mental arithmetic task as a function of the type of irrelevant sound in Experiment 1. Error bars depict standard errors of the mean

Figure 1 illustrates the average accuracy in the serial recall and mental arithmetic trials. As can be seen, performance on the serial recall task was lower in case of changing-state sound than in case of steady state sound (Fig. 1A), whereas this effect appears to be absent in the mental arithmetic task (Fig. 1B). A 2 (state of sound: steady-state, changing-state) × 2 (deviant: absent, present) × 2 (task: serial recall, mental arithmetic) ANOVA revealed a significant main effect of task (and strong Bayesian evidence), *F*(1,30) = 22.47; *p* < .001; η^2^_G_ = 0.136; *BF*_*Incl*_ = 765.961, with higher overall accuracy in the mental arithmetic task (*M* = .778; *SE* = .020) than in the serial recall task (*M* = .657; *SE* = .024). In addition, there was a significant main effect for the state of sound, *F*(1,30) = 11.65; *p* = .002; η^2^_G_ = 0.019; *BF*_*Incl*_ = 13.916, with higher overall performance during steady-state sound (*M* = .739; *SE* = .019) than during changing-state sound (*M* = .697; *SD* = .019). Most importantly, the analysis also confirmed the task-specificity of the changing-state effect a significant interaction between task and state of sound, *F*(1,30) = 6.91; *p* = .013; η^2^_G_ = 0.019; *BF*_*Incl*_ = 7.645.

Additional follow-up analyses within each task revealed a significant and highly likely changing-state effect on serial recall, *F*(1,30) = 27.28; *p* < .001; η^2^_G_ = 0.074; *BF*_*Incl*_ = 1026.597, but not on performance in the mental arithmetic task, *F*(1,30) < 0.01; *p* > .99; η^2^_G_ < 0.01; *BF*_*Incl*_ = 0.177.

Interestingly, the 2 × 2 × 2 ANOVA did not reveal a significant deviation effect, *F*(1,30) = 0.98; *p* = .331; η^2^_G_ = 0.002; *BF*_*Incl*_ = 0.166, and there was also no modulation of the deviation effect by the type of task, *F*(1,30) < 0.01; *p* = .973; η^2^_G_ < 0.001; *BF*_*Incl*_ = 0.147. The ANOVA further revealed no significant three-way interaction, *F*(1,30) = 1.22; *p* = .279; η^2^_G_ = 0.002; *BF*_*Incl*_ = 0.115, and no interaction between state of sound and deviant, *F*(1,30) = 0.38; *p* = .543; η^2^_G_ < 0.001; *BF*_*Incl*_ = 0.177.

The follow-up analyses further revealed no significant deviation effects both in serial recall, *F*(1,30) = 1.13; *p* = .296; η^2^_G_ = 0.002; *BF*_*Incl*_ = 0.460, and in the mental arithmetic task, *F*(1,30) = 0.35; *p* = .561; η^2^_G_ = 0.002; *BF*_*Incl*_ = 0.188. Moreover, in contrast to the descriptive trend (see Fig. 1A), serial recall was also not subject to a significant state × deviant interaction, *F*(1,30) = 2.66; *p* = .114; η^2^_G_ = 0.006; *BF*_*Incl*_ = 1.087. Pairwise comparisons (corrected for multiple comparisons; Benjamini & Hochberg, 1995) revealed that the deviation effect was non-significant both in steady-state (*p* = .65) and in changing-state (*p* = .07) sound. There was also no state × deviant interaction on mental arithmetic performance, *F*(1,30) = 0.05; *p* = .820; η^2^_G_ < 0.001; *BF*_*Incl*_ = 0.042.

### Discussion

Experiment 1 demonstrated that task-irrelevant changing-state sound disrupted serial recall, whereas the same sounds did not affect performance on a mental arithmetic task. Interestingly, Experiment 1 did not reveal a deviation effect in either task (though there is a small trend for a deviation effect with changing-state sequences on serial recall). Hence, in contrast to several previous observations (Banbury & Berry, 1998; Hadlington et al., 2006; Perham et al., 2013, 2016; Perham & Macpherson, 2012), there was no indication of auditory distraction of mental arithmetic performance in the present study. A possible reason for this could be the use of different types of distractors. Most previous studies have shown that mental arithmetic performance was impaired by the presence of speech (e.g., spoken numbers or letters) or continuous office noise with speech, compared to silence or office noise without speech (Banbury & Berry, 1998; Hadlington et al., 2006; Perham et al., 2016; Perham & Macpherson, 2012), but they did not contrast conditions of changing-state and steady-state speech as in the present study. Therefore, it could be argued that the disruptions of mental arithmetic in the previous studies may have been due to attentional capture rather than due to interference-by-process produced by the changing-state nature of speech. To disentangle the two processes, changing-state effect was contrasted with the deviation effect. Surprisingly, a single deviant voice was found to have no statistically significant disruptive effect on either task, suggesting that the deviant may not have elicited an attentional orienting response that was strong enough to disrupt either serial recall or mental arithmetic performance. Interestingly, the Bayesian statistics further revealed that it is somewhat more likely based on the present data that there is a deviation effect in both tasks than there being no effect. It could be argued that the types of speech sounds presented in the previous studies (e.g., ascending two-digit numbers; Perham et al., 2016; or numbers similar to the numbers used in the mental arithmetic task; Perham & Macpherson, 2012) have been more effective capturers of attentional resources and thus have disrupted mental arithmetic performance when compared to silence.

The results of Experiment 1 suggest that task-irrelevant changing-state sound produces specific interference with a serial short-term memory task, whereas it does not disrupt a presumably non-serial mental arithmetic task consisting of successive addition and subtraction problems. This finding is consistent with the assumption of the changing-state effect to be specific to serial-order processing (Jones et al., 1996). However, as there was no clear indication of an auditory deviation effect in either task, the results are ambiguous with regard to the mechanisms distinguishing the changing-state effect from the deviation effect (Hughes, 2014).

A reason for the absence of the deviation effect might be that the effect is smaller in fact than expected (based on previous reports; Hughes et al., 2007, Exp. 1). In addition, the sensitivity of the mental arithmetic task (producing either a true or a false response on each trial) is certainly lower than the sensitivity of the serial recall task (producing eight true or false responses on each trial), and therefore an equal number of trials in the mental arithmetic task (as in the serial recall task) may not have been sufficient. Therefore, a second experiment was conducted with enhanced statistical power and a mental arithmetic task producing several responses on each trial.

## Experiment 2

The aim of Experiment 2 was to replicate the task-specificity of the changing-state effect to serial recall, as compared to performance in a mental arithmetic task. In addition, it was investigated whether performance in both tasks was disrupted equally by the presence of an auditory deviant, which is expected to cause a diversion of central attention from the focal task (in line with attentional account and the duplex-mechanism account).

To increase the likelihood of finding an effect of auditory distracters in the mental arithmetic task, the statistical power was increased and the working-memory load imposed by the arithmetic task was increased by presenting more than one arithmetic problem on each trial (more similar to the serial recall task), while conducting a secondary task. Specifically, four math equations were presented, each requiring a validation response. The equations involved both the multiplication of two digits and the addition of a third digit that always exceeded the tens boundary, thus minimizing the likelihood of serial rehearsal strategies (e.g., counting) being used to evaluate the equation (see Perham et al., 2013, p. 144). In addition, to rule out the possibility that the task in Experiment 1 may have been insensitive to distraction because it was too easy, participants’ working memory capacity was demanded by simultaneously remembering word-pairs for later cued recall test (i.e., similar to an operation span task to measure working memory capacity; Turner & Engle, 1989). In contrast to Experiment 1, the serial recall and mental arithmetic tasks were presented to different groups of participants (a between-subjects design), thus ruling out possible carry-over effects due to differences in cognitive demand between the tasks.

One possible explanation for the absence of a deviation effect in the serial recall task of Experiment 1 might be the relatively slow presentation rate of the to-be-remembered items (2 s/item, which had been matched to the presentation rate in the mental arithmetic task) compared to previous studies (e.g., 1 s/item; Bell, Mieth, et al., 2019; or 750 ms/item; Hughes et al., 2005; Marois et al., 2019). Even if the auditory deviant captured attention, the long presentation time may have given participants the time to redirect attention back to the focal task before the next digit was presented. To test whether this can explain the absence of a deviation effect, a faster presentation rate (1.2 s/item) was used in Experiment 2.

In addition, due the lockdown of the laboratory during the COVID-19 pandemic, Experiment 2 had to be conducted using an online task environment. In order to keep the duration of the experiment as short as possible for each participant (and to minimize the risk of dropout), the task (serial recall vs. mental arithmetic) was manipulated between subjects. A quiet control condition was added to the experimental design allowing us to get some indication of whether or not individual participants had taken off the headphones during the task by observing a difference in serial recall performance between sound and quiet conditions. In addition, a headphone screening test (Woods et al., 2017) was conducted prior to the actual experiment to ensure that participants were using headphones (and not loudspeakers) at least when starting the study.

### Method

#### Participants

One hundred and one participants were recruited for Experiment 2 (72 women and 29 men). Ages ranged between 18 and 70 years (*M* = 28.8; *SD* = 11.1). Participants were randomly assigned to either the serial recall task (*n* = 44; 30 women; *M*_age_ = 28.9; *SD*_age_ = 11.6) or the mental arithmetic task (*n* = 57; 42 women; *M*_age_ = 28.8; *SD*_age_ = 10.8). Due to the COVID-19 pandemic, the experiment was conducted as an online study using the PsyToolkit 3.1.1 programming environment (Stoet, 2010, 2017). Given that the effect sizes of the changing-state effect and the deviation effect were relatively small (or absent) in Experiment 1, the sample size was increased in Experiment 2. In addition, more participants were required in Experiment 2 because the task was manipulated between subjects. Based on a sensitivity analysis of statistical power (conducted in G*Power), the sample size was sufficient to detect even a small effect size of *f* = 0.16 for the crucial interaction term (sound × task) in a repeated-measures ANOVA with a statistical power of 90% (α = .05; correlation *r* = .5 among repeated measures; no sphericity correction). Thereby, the sample size of Experiment 2 is appropriate to detect even a small effect size of the interaction such as the recently reported for the modulation of the changing-state effect with the use of serial-rehearsal versus non-serial strategies in the serial recall task (*f* = .23; compare Hughes & Marsh, 2020; p. 436).

In the serial recall group, one participant reported a “minimal” hearing loss on the left ear, and one other participant reported to suffer from a tinnitus. In the mental arithmetic group, one participant reported a tinnitus, and two participants reported occasional or mild tinnitus. All remaining participants in both groups reported normal hearing. The data of all participants were included in the analyses. The ethics committee of the Technical University of Darmstadt approved the protocol of Experiment 2 on 5 May 2020 (EK 20/2020). Student participants were compensated with partial course credit.

#### Apparatus

The routines for online stimulus presentation and response registration were programmed in PsyToolkit syntax (Stoet, 2010, 2017). Participants were able to take part in the study using Mozilla Firefox, Google Chrome, Microsoft Edge, and Opera as compatible browsers (it was not possible with Safari and Internet Explorer). Participants were instructed to wear headphones for the duration of the study.

#### Irrelevant sound

The same set of spoken consonants as in Experiment 1 was presented in Experiment 2, and the irrelevant auditory sequences were created in the same way, except for a few changes. Each recording of a consonant was cut to a duration of 1 s, and both steady-state and changing-state sequences were spoken by the female voice. To keep the number of distracter sounds in the mental arithmetic task (sequences 24 consonants) comparable to Experiment 1, the presentation rate of to-be-ignored consonants was slightly reduced in Experiment 2 (1 s/consonant). In the deviant sound conditions, individual consonants were replaced by the same consonant spoken by the male voice. In order to allow the auditory deviants to affect the evaluation of multiple arithmetic equations per trial, the number of deviants within a trial was varied randomly between one and three, and each deviant was presented (or not) during a different math equation.^1^ For the serial recall task (16 s trial duration), auditory sequences consisted of 16 spoken consonants, and for the mental arithmetic task (24 s trial duration), sequences consisted of 24 spoken consonants.

#### Experimental design

A 2 (task: serial recall, mental arithmetic) × 2 (state of sound: steady-state, changing state) × 2 (deviant: present, absent) mixed-variables design with state of sound and deviant as repeated-measures variables and task as a group variable was implemented. In addition to the sound conditions from Experiment 1, there was also a quiet control condition in Experiment 2. The sound conditions quiet, steady-state, and changing-state were each repeated 20 times, including five trials (25%) with a voice deviant in case of steady-state and changing-state sound.

#### Procedure

The experiment started with a *headphone-screening test* to make sure that participants wore headphones and set the volume to an appropriate level (Woods et al., 2017). Prior to this test, continuous pink noise was presented, and participants were instructed to wear headphones and to adjust the volume of their computer to a comfortable level. The RMS of the noise signal was 0.10. The headphone-screening test itself was a three-alternative forced-choice task with three 200-Hz tones being presented successively. Each tone was presented in stereo for 1 s (together with a turquoise square in the center of the screen), followed by a 200-ms inter-stimulus interval (and a blank screen). The level of one tone was 6 dB lower than the other two tones. One of the two high-intensity tones had the phase reversed between the left and right channels (in case of loudspeakers, this phase reversal was expected to reduce the sound pressure level in air, thus making it more difficult to detect the low-intensity tone; see Woods et al., 2017). After the third tone, participants were asked to indicate which of the three tones was softer than the other two by pressing the respective number key on their keyboard. The headphone test was passed if at least five responses were correct within a six-trial block. If less than five responses were correct, the test continued with another six-trial block until either five or six correct responses were made within a block. If participants did not pass the test within ten blocks, a message was shown on the screen, telling the participant that the study was terminated because the audio system was not sufficient to proceed. Otherwise, the experiment continued with either the serial recall or mental arithmetic task.

Both tasks then started with four randomly chosen practice trials, followed by 60 experimental trials. Each trial started with a 1-s preparation cue in which a turquoise square decreasing in size was presented in the center of the black screen (full screen mode).

In the *serial recall task*, eight randomly drawn digits were then presented successively in white font for 1 s each. The digits were separated blank inter-stimulus intervals of 200 ms. After the last digit, there was an additional 6.6-s retention interval with a blank screen before participants were asked to recall the digits in order. Therefore, a text prompt was shown on the screen and participants could type the digits using the keyboard, without an option to correct. After the eighth entered response, feedback was presented for 1.5 s, showing the number of digits that were recalled in the correct serial position. Irrelevant sound was played for 16 s, during both the presentation of digits and the retention interval (except on quiet trials).

In the *mental arithmetic task*, four math equations (e.g., “(5 × 6) + 7 = 35”) together with four German word pairs (e.g., “Baum – Sinn”) were presented on the black screen for 5 s each. The equation was presented in yellow font in the center of the screen, and the word pair were presented on top of the equations in cyan font. The word pairs were selected randomly without replacement on each trial from a set of eighteen neutral German monosyllabic words (“Alm”, ”Baum”, “Chor”, “Ding”, “Elch”, “Fang”, “Gurt”, “Helm”, “Kamm”, “Los”, “Mund”, “Norm”, “Post”, “Riss”, “Sinn”, “Tuch”, “Volt”, and “Zaun”). The math equations consisted of two additively combined terms on the left side of the equality sign, with the first term consisting of two multiplicatively combined numbers between 1 and 9, and the second term being a single number between 1 and 9. The right side of the equality sign was either the correct solution (in 50% of the trials) or a number that differed from the correct solution by -2, -1, 1, or 2. Participants were asked to indicate whether the equation was correct or wrong within the 5 s of presentation time by pressing the “J” (yes) or “N” (no) key, respectively. In addition, participants were required to remember the word pairs for later recall, regardless of the order in which the word pairs were presented. After each equation, feedback was presented for 1 s, with a green circle indicating that the response to the math equation was correct, and a red circle indicating that the response was incorrect. After the feedback to the fourth math equation (i.e., after 24 s), the first word of one of the four word pairs (randomly chosen) was presented on the screen and participants were asked to recall the associate word of that pair by typing it in. At the end of each trial, overall feedback was presented for 2.5 s, indicating whether the word was recalled correctly or not, and the number of correctly judged math equations. The next trial started after an inter-trial interval of 500 ms.

At the end of the experiment, participants of both groups were asked to indicate what strategies they used to perform the task (i.e., to memorize the order of digits or evaluate the math equations while remembering word pairs, respectively). It was an open question and the answers could be typed in a text box. On average, the entire experiment (including the headphone screening test) took 43 min.

### Results

**Fig. 2:**
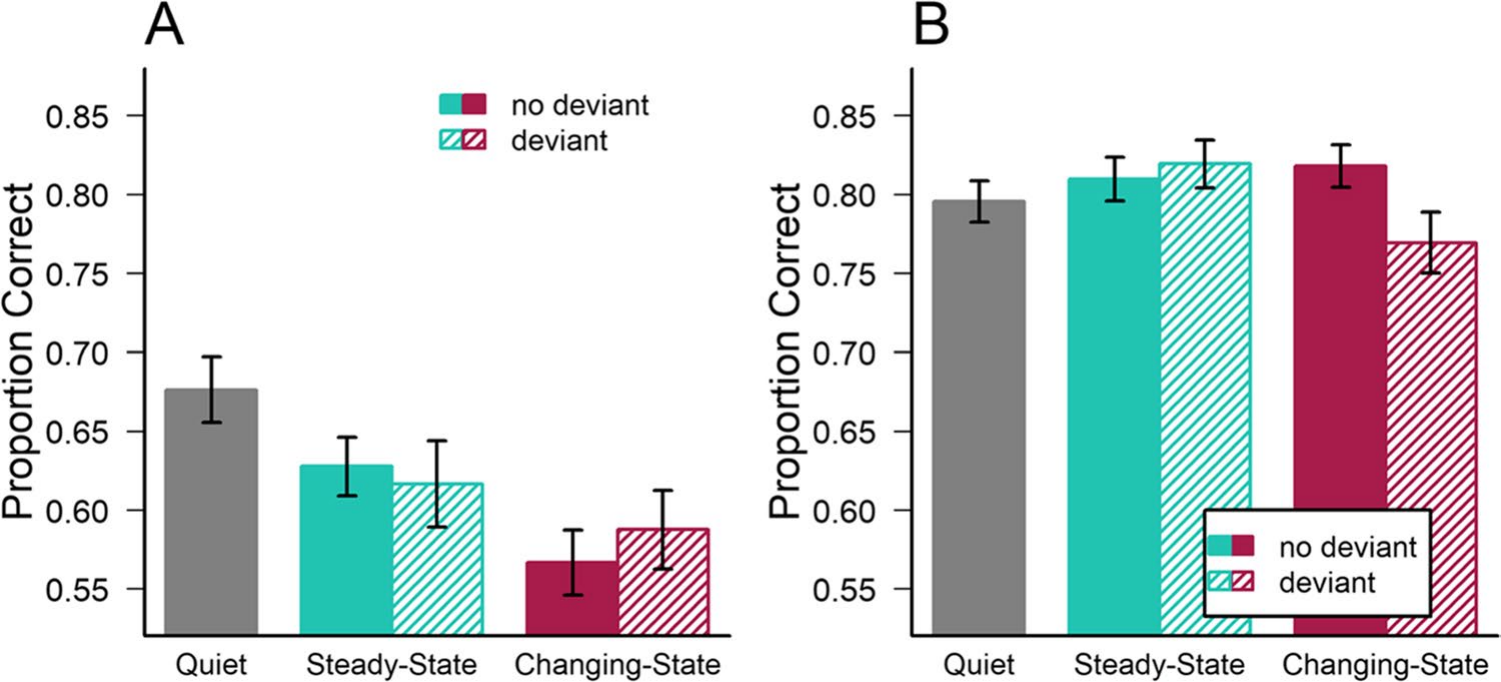
Mean accuracy in (**A**) the serial recall (eight digits per trial) and (**B**) the mental arithmetic and word pair memory task (evaluations of four arithmetic equations per trial) as a function of the type of irrelevant sound in Experiment 2. Error bars depict standard errors of the mean

The average accuracy in the serial recall and the mental arithmetic tasks under the different sound conditions is illustrated in Fig. 2 (for the arithmetic task, accuracy corresponds to the average number of correct evaluations of math equations, i.e., is based on four responses per trial). As can be seen, serial recall was clearly impaired by the presence of changing-state sound, as compared to both steady-state sound and quiet (Fig. 2A). In contrast, no general changing-state effect was evident in the mental arithmetic task (Fig. 2B). However, in contrast to Experiment 1, an auditory deviant in changing-state sound appears to have reduced performance on the mental arithmetic task.

First, to test for task differences in *steady-state and changing-state effects*, a 2 (task: serial recall, mental arithmetic) × 3 (sound: quiet, steady-state, changing-state; all without deviants) mixed-factors ANOVA with task as a between-subjects factor and sound as a repeated-subjects factor was conducted. The analysis revealed a significant (and extremely likely) main effect of task, *F*(1,99) = 43.06; *p* < .001; η^2^_G_ = 0.271; *BF*_*Incl*_ > 10^14^, a main effect of sound, *F*(2,198) = 17.87; *p* < .001; η^2^_G_ = 0.026; *BF*_*Incl*_ > 10^11^, as well as a sound × task interaction, *F*(2,198) = 33.01; *p* < .001; η^2^_G_ = 0.046; *BF*_*Incl*_ > 10^10^. Individual comparisons corrected for multiple comparisons (according to Benjamini & Hochberg, 1995) revealed significant differences between all three sound conditions in serial recall (*p* < .001), i.e., both a steady-state and a changing-state effect, whereas there was only a significant contrast between quiet and changing-state sound (*p* = .03) in the mental arithmetic task (*p* = .14 and *p* = .33 for the two other contrasts, respectively).

Second, testing for *deviation effects* in the two tasks, a 2 (task) × 3 (sound: quiet, sound without deviant, sound with deviant; collapsed across steady-state and changing-state conditions) mixed-factors ANOVA revealed a main effect of task, *F*(1,99) = 36.92; *p* < .001; η^2^_G_ = 0.241; *BF*_*Incl*_ > 10^10^, a main effect of sound, *F*(2,198) = 13.87; *p* < .001; η^2^_G_ = 0.020; *BF*_*Incl*_ > 10^6^, as well as a sound × task interaction, *F*(2,198) = 17.14; *p* < .001; η^2^_G_ = 0.025; *BF*_*Incl*_ = 310304.842. For serial recall, corrected pairwise comparisons revealed a significant contrast between quiet (*M* = 0.68; *SD* = 0.14) and sounds with (*M* = 0.60; *SD* = 0.15; *p* < .001) or without deviant (*M* = 0.60; *SD* = 0.12; *p* < .001), but there was no difference between sounds with and without deviant (*p* = .75), i.e., no deviation effect. In contrast, performance in the mental arithmetic task was subject to a significant deviation effect (sounds without deviant, *M* = 0.81; *SD* = 0.10 vs. sounds with deviant, *M* = 0.79; *SD* = 0.12; *p* = .047), a significant difference between sounds without a deviant and quiet (*M* = 0.80; *SD* = 0.10; *p* = .047), but no significant difference between sounds with deviants and quiet (*p* = .90; note that arithmetic performance during quiet was worse than during sounds without a deviant, see Fig. 2).

Third, to test for possible *interactions between deviation and changing-state effects*, a 2 (task) × 2 (state of sound) × 2 (deviant) mixed-factors ANOVA with state of sound and deviant as repeated-measures factors was conducted after exclusion of the quiet trials. This analysis again revealed a significant main effect of task, *F*(1,99) = 48.77; *p* < .001; η^2^_G_ = 0.270; *BF*_*Incl*_ > 10^7^, with lower accuracy in the serial recall task (*M* = 0.600; *SE* = 0.012) than in the mental arithmetic task (*M* = 0.762; *SD* = 0.015). In addition, there was also a main effect of the state of sound, *F*(1,99) = 12.97; *p* < .001; η^2^_G_ = 0.011; *BF*_*Incl*_ = 7.685, with impaired performance during changing-state (*M* = 0.667; *SE* = 0.012) compared to steady-state sound (*M* = 0.695; *SD* = 0.012). Importantly, the ANOVA also revealed a significant interaction between task and the state of sound *F*(1,99) = 4.94; *p* = .028; η^2^_G_ = 0.004; *BF*_*Incl*_ = 2.358. There was no significant main effect of the presence of a deviant, *F*(1,99) = 0.56; *p* = .456; η^2^_G_ < 0.001; *BF*_*Incl*_ = 0.117, and no significant interaction between deviant and task, *F*(1,99) = 1.95; *p* = .166; η^2^_G_ = 0.002; *BF*_*Incl*_ = 0.197. However, there was a significant three-way interaction (but inconclusive Bayesian evidence), *F*(1,99) = 5.302; *p* = .023; η^2^_G_ = 0.005; *BF*_*Incl*_ = 0.472, indicating that deviants in changing-state sound may be more disruptive of mental arithmetic performance than deviants in steady-state sound – i.e. a combination of the disruptive effects of changing-state sound and a voice deviant seems to affect mental arithmetic performance (compare Fig. 2B).

To further decode the three-way interaction, separate 2 (state of sound) × 2 (deviant) repeated-measures ANOVAs were conducted for participants in the serial recall and mental arithmetic groups. For serial recall there was a significant main effect of the state of sound, *F*(1,43) = 10.45; *p* = .002; η^2^_G_ = 0.022; *BF*_*Incl*_ = 6.040, with changing-state sound disrupting recall performance (*M* = 0.577; *SE* = .021) to a greater extent than steady state sound (*M* = 0.622; *SE* = 0.021). However, there was no deviation effect, *F*(1,43) = 0.13; *p* = .720; η^2^_G_ < 0.001; *BF*_*Incl*_ = 0.193, and no interaction, *F*(1,43) = 1.29; *p* = .262; η^2^_G_ = 0.003; *BF*_*Incl*_ = 0.283. In contrast, there was no significant changing-state effect on performance in the mental arithmetic task, *F*(1,56) = 1.643; *p* = .205; η^2^_G_ = 0.002; *BF*_*Incl*_ = 0.33. Interestingly, there was a non-significant trend for a deviation effect, *F*(1,56) = 3.90; *p* = .053; η^2^_G_ = 0.005; *BF*_*Incl*_ = 1.316 (without deviant: *M* = 0.770; *SE* = 0.013; with deviant: *M* = 0.754; *SE* = 0.015), as well as a significant interaction on mental arithmetic performance, *F*(1,56) = 5.44; *p* = .023; η^2^_G_ = 0.008; *BF*_*Incl*_ = 1.921, indicating that the deviation effect was actually restricted to the changing-state sound condition (see Fig. 2B).

In the analyses above, performance on the mental arithmetic task was restricted to the evaluation of math equations. To enhance the task demand, participants in the mental arithmetic group were asked to simultaneously memorize a word pair while evaluating the math equations. The average *recall accuracy for the word pairs* is shown in Table 1. A 2 (state of sound) × 2 (deviant) repeated-measures ANOVA on pair-associate memory revealed no significant changing-state effect, *F*(1,56) = 2.33; *p* = .133; η^2^_G_ = 0.005; *BF*_*Incl*_ = 0.312, no deviation effect, *F*(1,56) = 0.01; *p* = .910; η^2^_G_ < 0.001; *BF*_*Incl*_ = 0.128, and no interaction, *F*(1,56) = 0.35; *p* = .556; η^2^_G_ < 0.001; *BF*_*Incl*_ = 0.043, indicating that pair-associate memory during the mental arithmetic task was not susceptible to auditory distraction at all.

**Table 1:** Mean accuracy of pair-associate memory (word pairs) as a function of the type of irrelevant sound (SS = steady-state; CS = changing-state) presented during the mental arithmetic task of Experiment 2

Sound condition	*M*	*SD*
Quiet	0.611	0.186
SS	0.586	0.201
SS + deviant	0.572	0.260
CS	0.605	0.182
CS + deviant	0.614	0.229

The answers given to the open task-strategy question at the end of the experiment were categorized (by the first author) into different types of strategies (similar to Morrison et al., 2016). In the serial recall group, most participants reported a strategy that was categorized either as “rehearsal” (*n* = 19; e.g., “silent repetition of the digits”) or as “grouping” (*n* = 12; e.g., “think of two-digit numbers”), and these two types of strategies were considered as “serial-processing” strategies (in line with Hughes & Marsh, 2020). Other strategies included “visual imagery” of the digits (*n* = 7), “singing” (*n* = 4), “semantic associations” (*n* = 3), and “concentrate on the first/last digits” (*n* = 6). In total, 30 participants reported a serial-processing strategy, and only 14 participants reported only non-serial strategies (note that several participants reported multiple strategies). In the mental-arithmetic group, most participants reported to have used “visual imagery” as a strategy to remember the word pairs (*n* = 22), followed by “rehearsal” (*n* = 20), and “semantic association” strategies (*n* = 10). Other strategies to remember the word pairs included “remember the initials” (*n* = 2) and “familiarity of the words” (*n* = 1). Only very few participants reported a mental-arithmetic strategy, and these strategies included “estimation” (*n* = 6), “concentrate on the equations” (*n* = 2), and “guessing” (*n* = 3). In total, 35 participants reported a non-serial strategy (e.g., imagery) and 21 participants reported a serial-rehearsal strategy (only to remember the word pairs) for the mental arithmetic task.

To test whether auditory distraction of serial recall depended on the *processing strategy* (and in particular whether the changing-state effect was restricted to participants who engaged in serial-order processing), separate 2 × 2 repeated-measures ANOVAs on *serial recall* performance revealed a significant changing-state effect in participants who reported a serial-order processing strategy, *F*(1,29) = 18.77; *p* < .001; η^2^_G_ = 0.042; *BF*_*Incl*_ = 42.831, but not in participants who reported a non-serial strategy, *F*(1,13) = 0.13; *p* = .725; η^2^_G_ = 0.001; *BF*_*Incl*_ = 0.380. In both groups, there was no significant deviation effect, *F*(1,29) = 0.43; *p* = .516; η^2^_G_ = 0.001; *BF*_*Incl*_ = 0.243 and *F*(1,13) = 2.81; *p* = .118; η^2^_G_ = 0.014; *BF*_*Incl*_ = 0.536, respectively, and no interaction, *F*(1,29) < 0.01; *p* = .955; η^2^_G_ < 0.001; *BF*_*Incl*_ = 0.234 and *F*(1,13) = 2.56; *p* = .134; η^2^_G_ = 0.023; *BF*_*Incl*_ = 0.618, respectively.

Interestingly, for the *mental-arithmetic task*, there was a significant changing-state effect, *F*(1,35) = 5.45; *p* = .025; η^2^_G_ = 0.012; *BF*_*Incl*_ = 9.308, a significant deviation effect, *F*(1,35) = 5.01; *p* = .032; η^2^_G_ = 0.011; *BF*_*Incl*_ = 8.414, as well as a significant interaction, *F*(1,35) = 7.35; *p* = .010; η^2^_G_ = 0.018; *BF*_*Incl*_ = 23.280, in participants who reported to have *not* used serial rehearsal as a strategy to memorize the word pairs (see Table 2). In contrast, mental arithmetic performance in participants who reported to have used serial rehearsal to memorize the word pairs, was not susceptible to auditory distraction, i.e., there was no changing state effect, *F*(1,20) = 0.99; *p* = .332; η^2^_G_ = 0.003; *BF*_*Incl*_ = 0.310, no deviation effect, *F*(1,20) = 0.63; *p* = .436; η^2^_G_ = 0.002; *BF*_*Incl*_ = 0.298, and no interaction, *F*(1,20) = 2.20; *p* = .153; η^2^_G_ = 0.011; *BF*_*Incl*_ = 0.347.

**Table 2:** Mental arithmetic accuracy (evaluation of math equations) in participants who reported to have used serial rehearsal to memorize the word pairs and in participants who did not engage in rehearsal during the task under different sound conditions (SS = steady-state; CS = changing-state)

Sound condition	Serial rehearsal (*n* = 21)		No serial rehearsal (*n* = 36)	
	*M*	*SD*	*M*	*SD*
SS	0.798	0.027	0.816	0.093
SS + deviant	0.812	0.117	0.824	0.114
CS	0.810	0.118	0.822	0.094
CS + deviant	0.774	0.135	0.767	0.154

### Discussion

The task-specificity of the disruptive effects produced by changing-state sound was replicated in Experiment 2, revealing once more a clear changing-state effect on serial recall, but not on performance in the mental arithmetic task. Hence, a general changing-state effect was not observed even with a more sensitive (i.e., multiple responses per trial) and more demanding mental arithmetic task requiring four math equations to be evaluated successively on each trial. In addition, and consistent with a recent large-scale investigation (Bell, Röer, et al., 2019a), steady-state speech was found to produce reliable disruption of serial recall, compared to a quiet control condition (i.e., a steady-state effect). The fact that in particular the changing-state effect could be demonstrated in an online experiment (allowing much less control over the exact stimulus parameters) suggests that this type of auditory distraction is a robust and ecologically valid phenomenon. Furthermore, the demonstration of a steady-state effect is largely compatible with a graded attentional account of auditory distraction assuming that even repeated sounds may produce small attentional capture effects (see Bell et al., 2012), whereas the observation of a steady-state effect is more difficult to explain with interference-by-process (Hughes, 2014). Nevertheless, an attentional account of auditory distraction would predict similar disruptive effects of steady- and changing-state sound in other cognitive tasks as well. The fact that performance on the mental arithmetic task was immune to these two effects is more in line with the assumption of specific interference with serial-order processing. Hence, without further assumptions, the present findings are not entirely compatible with any of the accounts.

Moreover, and in contrast to Experiment 1, the presence of an auditory deviant in the sequence of irrelevant changing-state speech (i.e., an unexpected change of voice) was found to disrupt performance in the mental arithmetic task, whereas it did again not affect serial recall. Interestingly, the deviation effect on mental arithmetic was restricted to the changing-state background sound, but a deviant voice in a steady-state sequence did not disrupt the evaluation of math equations. This unexpected finding suggests that a combination of changing-state sound and a deviant stimulus is required to impair mental arithmetic performance, whereas the two effects alone do not seem to be strong enough to produce disruption in this task. Specifically, disruption of performance in a mental arithmetic task seems to be susceptible to both the interference of changing-state sound with the amount of serial-order processing required for the task (e.g., brief rehearsal of the numbers on the left-hand side of the equation) and attentional capture by deviant sounds. However, both effects alone do not seem to be strong enough to impair performance, but the two effects together caused reliable distraction. Alternatively, it could be argued in line with an attentional account (e.g., Cowan, 1995) that distraction in the changing-state condition with a deviant stems from a combination of two sources of attentional capture (diversion of attention by changing phonemes and to a change in voice quality). Interestingly, an additional analysis of the cognitive strategies used during the task revealed that the susceptibility of mental arithmetic performance to auditory distraction (i.e., produced by both changing-state sound and an auditory deviant together) was restricted to participants who did not use serial rehearsal to memorize the word pairs while performing the mental arithmetic task. This observation suggests that the changing-state sound must indeed have caused some interference with the processing of the math equations.

Comparing the results of Experiments 1 and 2 further suggests that the more demanding mental arithmetic task (combined with a pair-associate memory task) was more susceptible to distraction by a combination of auditory deviants with changing-state sound than the less demanding addition and subtraction task. This observation appears to be inconsistent with the prediction of the duplex-mechanism account that an increase in task load should reduce the deviation effect (see Hughes et al., 2013). On the other hand, the average accuracy in the more demanding (dual-task) mental arithmetic task in Experiment 2 (80%) was even slightly higher than in the “simple” addition and subtraction task of Experiment 1 (78%), indicating that the task load in Experiment 2 might not have been higher than in Experiment 1.

Interestingly, neither changing-state sound nor an auditory deviant impaired the recall of pair-associate words (note that there was even a trend of pair-associate memory to be slightly enhanced by changing-state sound compared to steady-state sound, see Table 1), whereas previous studies did observe an irrelevant speech effect on pair-associate memory (Beaman & Jones, 1997; Exp. 4). This discrepancy might be due to the fact that participants in the present study did not recall the word pairs very well in the first place (they seem to have prioritized the evaluation of math equations), but it might also suggest that serial rehearsal has not been used as the predominant strategy to memorize the word-pairs in the current dual-task context.

Taken together, the findings of Experiment 2 are consistent with the assumption that changing-state sound produces specific interference with serial-order processing (Jones et al., 1996), as required for the recall of a series of digits. In contrast, performance in a mental arithmetic task, which is unlikely to involve serial-order processing, was not affected by the presence of changing-state sound (thus replicating Experiment 1). The presence of an auditory deviant, on the other hand, appears to have diverted attentional resources leading to impaired performance in a cognitively demanding non-serial task such as practicing mental arithmetic, whereas it did not affect serial recall. The fact that a deviation effect was observed only when changing-state sound was presented in the background (or that a changing-state effect was observed only when a deviant was present), suggests that a combination of the two types of auditory distraction may have been essential to cause disruption of mental arithmetic performance (while each effect alone may have been too small to cause reliable disruption), which is consistent with the unitary attentional account (Bell et al., 2012; Cowan, 1999) assuming both effects to be based on essentially the same mechanism (attentional capture).

Nevertheless, the absence of a deviation effect in the serial recall task is clearly at odds with several previous studies (e.g., Bell, Mieth, et al., 2019; Hughes et al., 2005, 2007; but see Kattner & Bryce, 2022), and it is not predicted by either the unitary attentional account nor the duplex-mechanism account. In addition, it needs to be noted that many procedural characteristics differed between Experiments 1 and 2 (e.g., lab vs. online study, duration of distracter sounds, presentation rate of digits and math equations, retention interval, gender of deviant voice). Therefore, a third experiment was conducted to bridge the gap between the two experiments by using procedural properties from Experiment 1 and a different strategy to increase the difficulty of the mental arithmetic task. In addition, some aspects were matched more closely with the procedures of previous studies that observed a deviation effect (e.g., the exact position of the deviant).

## Experiment 3

The aim of Experiment 3 was to provide additional support for the assumption that changing-state sound produces disruption in serial recall but not in a mental arithmetic task, using slightly different procedural properties. Specifically, a fast presentation rate (1 s / item) was used both for serial recall and an adapted mental arithmetic task. In addition, in order to enhance the likelihood of observing a strong deviation effect not only in the mental arithmetic but also in the serial recall task, additional constraints were applied to the exact sequences of distracter sounds. One issue might be the exact position of the deviant relative to the sequence of to-be-remembered items. In Experiment 1, the deviant occurred together with the fourth digit (both in the serial recall and mental arithmetic task), whereas in Experiment 2, multiple deviants were presented at unpredictable positions together with up to three math equations on a given trial (a rather unusual procedure, which was chosen to give deviants the chance to affect the evaluations of more than one math equation). To be more consistent with the procedure of previous studies, the deviant was presented as the seventh irrelevant letter starting 100 ms before the presentation of the fifth relevant item in Experiment 3 (compare Hughes et al., 2005; p. 1053; see also Kattner & Bryce, 2022, Exp. 4).

In addition, as in Experiment 1 (but in contrast to Experiment 2), the task was manipulated within subjects. However, to avoid carry-over effects between the different tasks (e.g., due to different cognitive demands) participants completed the serial recall and the mental arithmetic tasks in two separate blocks, and the length of the two tasks were matched, thus also eliminating a possible confounding between type of task and the total exposure to irrelevant sound (i.e., the “token dose”; Bridges & Jones, 1996). Finally, in addition to both previous experiments, participants were asked to indicate their recall strategy after the serial recall task, allowing both the changing-state effect and the deviation effect to be analyzed separately as a function of whether or not participants engaged in serial rehearsal. Specifically, the changing-state effect is expected to disrupt serial recall only when participants engage in serial-order processing, whereas the deviation should affect serial recall regardless of the particular recall strategy.

### Method

#### Participants

Seventy-seven English-speaking participants were recruited via Prolific (https://app.prolific.co/), and the data of 72 participants, who completed the task properly and without technical problems, were included in the analyses (24 female, 46 male, two non-binary). The sensitivity analysis (conducted with G*Power) revealed that this sample size was sufficient to demonstrate an interaction (between sound and task) with an effect size of *f* = .30 or larger in a repeated-measures ANOVA with a statistical power of 90% (α = .05, correlation *r* = .5 among repeated measures, no correction of sphericity).

No pre-screening criteria were applied in Prolific (“standard sample” option), but participants were informed that proficiency in English was required to complete the task. In addition, participants were instructed to participate only if they were able to work on the task at a quiet place and without interruption to be expected for about 45 min. Ages ranged between 18 and 72 years (*M* = 29.9; *SD* = 12.1 years). All participants were compensated with £7.50/h. Based on the IP addresses, most participants were located in the United Kingdom (*n* = 19), Poland (*n* = 15), the United States (*n* = 9), Greece (*n* = 7), or Italy (*n* = 5). The remaining participants (each *n* < 5) were located in Chile, Latvia, Finland, France, Romania, Portugal, Israel, Germany, Austria, the Netherlands, and Mexico. Five participants did not complete the task properly, so the final sample consisted of 70 participants.

#### Apparatus and irrelevant sound

Both tasks of Experiment 3 were programmed in PsyToolkit syntax (Stoet, 2010, 2017), and participants conducted the tasks on their own computers using Mozilla Firefox, Google Chrome, Microsoft Edge, or Opera as web browsers. Headphones should be worn throughout the experiment.

The steady-state and changing-state sound sequences with (15 each) and without (five each) deviant were created in Python and then uploaded to the PsyToolkit server. Each sequence consisted of 15 700-ms recordings of letters spoken by a female voice, thus generating a total sequence length of 10.5 s. In steady-state sequences one letter was drawn randomly (from ten unique letters: b, f, g, k, l, m, n, p, s, t) and repeated 15 times, and in changing-state sequences the 15 letters were drawn randomly with replacement from the ten unique letters. In sequences with a deviant, the seventh letter was replaced by the same letter spoken by the male voice (i.e., the deviant started after 4.9 s, i.e., 100 ms prior to the onset of the fifth to-be-remembered item).

#### Experimental design

A 2 (task: serial recall, mental arithmetic) × 2 (state of sound: steady-state, changing state) × 2 (deviant: present, absent) within-subjects design. In addition to the four irrelevant sound conditions, there was also a quiet control condition as in Experiment 2. The quiet, steady-state, and changing-state conditions were repeated 15 times (per task), and there were five additional steady-state and changing-state trials with a deviant voice, thus resulting in a total of 55 trials per task.

#### Procedure

Before the experiment, participants had to complete the same headphone-screening test as in Experiment 2, to ensure that they wore proper headphones. The test was passed when more at least five correct responses were given within a block of six trials, and participants were allowed to run a maximum of five blocks. If a participant did not pass the headphone-screening test, the experiment was ended, but the participant was informed that he or she could restart the experiment with a different audio equipment. If the headphone test was successful, then the participant completed both the serial recall and the mental arithmetic task. The order of the two tasks was determined randomly for each participant. Both tasks started with two practice trials (on in silence and one with steady-state sound in the background), which were not included in the analysis.

The serial recall task consisted of 55 trials. The participants clicked on a green “Go” button to start each trial. Then, after a 500-ms delay, nine digits from 1 to 9 were presented sequentially and in random order (without replacement) in white font on a black background. Each digit was presented for 750 ms and followed by a 250-ms inter-stimulus interval. The irrelevant sound started with the onset of the first digit and ended 1.5 s after the offset of the last digit (i.e., after 10.5 s). There were 15 trials with steady-state sound, changing-state sound and quiet, and additional five trials each with steady-state and changing-state sound containing a deviant voice, respectively. After the last digit, there was a 2.5-s retention interval before the numeric response matrix was shown on the screen together with a text prompt (“Please click the digits:”). Participants were now asked to enter the nine digits in the order of presentation, without the option to correct their responses. During the response, the sequence of clicked digits was presented below the response matrix. After the last response, text feedback was presented on the screen for 1,500 ms informing the participants about the number of correctly recalled digits (e.g., “5 of 9 correct!”).

The mental arithmetic task also consisted of 55 trials with the exact same sound conditions. As in the serial recall task clicking on a “Go” button started the presentation of nine items in white font on a black background. The items consisted of five digits and four arithmetic operators (+ or –), which were presented in alternations starting and ending always with a digit. On half of the trials, all arithmetic operators were “+” signs, and on half of the trials “–“ and “+” signs alternated (the procedure was adopted from Banbury & Berry, 1998). Each item (digit or operator) was presented for 750 ms and followed by a 250-ms inter-trial interval. The participants’ task was to add or subtract the numbers accordingly. After the last item, there was a 2,500-ms retention interval before participants were prompted to enter the result of the additions and subtractions in a text box (using the number keys on their keyboard). The response was confirmed with the ENTER key, and then short text feedback was presented on the screen for 1,500 ms, indicating on whether or not the result was correct (“Correct!” or “Wrong!”).

After the serial recall task, participants were asked to indicate the memorization strategy using a previously developed strategy questionnaire for memory tasks (Morrison et al., 2016). In this questionnaire, participants could check one or more of the following eleven options: (1) “I expected certain items to appear and mentally checked them off as they arrived” (checklist), (2) “I silently repeated the items” (rehearsal), (3) “I remembered the items in groups” (grouping), (4) “I thought about the way the items sounded” (sound), (5) “I answered based on what items seemed recent or familiar” (familiarity), (6) “I simply concentrated on the items” (concentrate), (7) “I created a visual image based on the meaning of the items” (imagery), (8) “I pictured the way the items looked on the screen” (look), (9) “I thought about other things that could relate to the items” (association), (10) “I used the meaning of the items to remember or connect them” (semantic), (11) “I used none of these strategies” (other). In line with a previous investigation of recall strategies in the context of the missing item task (Hughes & Marsh, 2020; Exp. 2, p. 436), the responses to this questionnaire were used to classify participants as having used a serial rehearsal strategy (either rehearsal or grouping or both) or a non-serial strategy to memorize the digits.

At the end of the experiments, participants were asked to respond to several additional questions to check whether they had received external help from another person or using paper and pencil, whether they had turned down the volume or taken off the headphones, whether they spoke aloud, whether they were distracted, and whether they switched to a different activity or browser during the task. On average, the entire experiment took 56 min.

### Results

None of the participants indicated that they had help from another person or that they used any external help such as paper and pencil during the task. All participants confirmed that they did not turn down the volume on their headphones during the task. Only one participant indicated to have taken off the headphones during the task. Fourteen participants reported to have spoken the digits aloud when trying to remember them. Five participants reported occasional distraction (“neighbor mowing grass,” “mom crossed the room,” “other people speaking in the room”).^2^ No participant reported to have switched to a different activity or browser during the task.

Based on the recall strategy questionnaire, most participants reported to have used “rehearsal” (*n* = 47) or grouping (*n* = 45) as a strategy in serial recall, and many participants reported both serial strategies together (*n* = 30). Other strategies, such as “concentrate” (*n* = 17), “look” (*n* = 17), “familiarity” (*n* = 11), or “imagery” (*n* = 9), were reported less frequently and often together with “rehearsal” and “grouping”. Altogether, 66 participants (91.7%) reported at least one serial strategy (“rehearsal” or “grouping”; Hughes & Marsh, 2020), and only six participants reported neither of these two strategies.

**Fig. 3:**
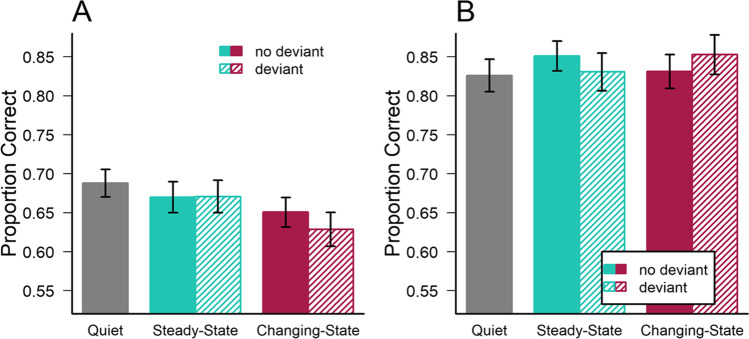
Mean accuracy in (**A**) the serial recall (nine digits per trial) and (**B**) the mental arithmetic task (additions and subtractions of five digits) as a function of the type of irrelevant sound in Experiment 3. Error bars depict standard errors of the mean

As in Experiment 2, task differences in *changing-state and steady-state effects* were first tested with a 2 (task) × 3 (sound: quiet, steady-state, changing-state) mixed-factors ANOVA on the accuracy in all trials without a deviant sound. The analysis revealed a significant main effect of task, *F*(1,71) = 67.24; *p* < .001; η^2^_G_ = 0.20; *BF*_*Incl*_ > 10^8^, with higher accuracy in the mental arithmetic task (*M* = .836; *SE* = .018) than in the serial recall task (*M* = .669; *SE* = .018). Interestingly, while the main effect of sound was not significant, *F*(2,142) = 2.50; *p* = .086; η^2^_G_ = 0.003; *BF*_*Incl*_ = 0.522, there was a significant interaction between sound and task, *F*(2,142) = 3.90; *p* = .002; η^2^_G_ = 0.004; *BF*_*Incl*_ = 1.729, indicating that the changing-state effect was restricted to serial recall (see Fig. 3).^3^

Task differences in terms of the *deviation effect* were analyzed with a 2 (task) × 3 (sound: quiet, without deviant, with deviant) mixed-factors ANOVA (i.e., the data were collapsed across steady-state and changing-state conditions). The analysis revealed a significant main effect of task, *F*(1,71) = 69.97; *p* < .001; η^2^_G_ = 0.21; *BF*_*Incl*_ > 10^9^, as well as a significant modulation of the deviation effect by task, *F*(2,142) = 5.42; *p* = .005; η^2^_G_ = 0.005; *BF*_*Incl*_ = 3.216. Adjusted post hoc comparisons revealed that the crucial difference between sounds with and without deviant was non-significant both in the serial recall (*p*_*holm*_ = .838) and the mental arithmetic task (*p*_*holm*_ = .958). Sounds with a deviant produced significant disruption of serial recall compared to quiet (*p*_*holm*_ = .015), but there was no disruption of mental arithmetic performance (*p*_*holm*_ = .838). The analysis of deviation effects revealed no main effect of the type of sound, *F*(2,142) = 0.77; *p* = .463; η^2^_G_ < 0.001; *BF*_*Incl*_ = 0.614.

Third, the *changing-state and deviation effects* in the two tasks were analyzed together using a 2 (task) × 2 (state of sound) × 2 (deviant) mixed-factors ANOVA on performance on all non-silent trials (note that the inclusion of task order as another between-subjects factor did not reveal any additional significant effects). The analysis revealed again a significant main effect of task, *F*(1,71) = 77.79; *p* < .001; η^2^_G_ = 0.208; *BF*_*Incl*_ > 10^9^, but no general changing-state effect (main effect of state of sound), *F*(1,71) = 2.26; *p* = .137; η^2^_G_ = 0.002; *BF*_*Incl*_ = 0.266, and no general deviation effect, *F*(1,71) = 0.34; *p* = .562; η^2^_G_ < 0.001; *BF*_*Incl*_ = 0.073. In addition, the task × state interaction did not reach statistical significance, *F*(1,71) = 3.35; *p* = .071; η^2^_G_ = 0.002; *BF*_*Incl*_ = 0.502, and there was also no task × deviant interaction, *F*(1,71) = 0.49; *p* = .487; η^2^_G_ < 0.001; *BF*_*Incl*_ = 0.076, and no interaction between state of sound and deviant , *F*(1,71) = 0.41; *p* = .524; *BF*_*Incl*_ = 0.037. However, the analysis revealed a significant three-way interaction, *F*(1,71) = 5.96; *p* = .017; η^2^_G_ = 0.002; *BF*_*Incl*_ = 0.080.

To decode the three-way interaction, two separate 2 (state) × 2 (deviant) ANOVAs were conducted on performance in the serial recall and the mental arithmetic task. Interestingly, performance in the *serial recall task* was subject to a significant changing-state effect, *F*(1,71) = 8.37; *p* = .005; η^2^_G_ = 0.008; *BF*_*Incl*_ = 4.353, but no deviation effect, *F*(1,71) = 1.12; *p* = .294; η^2^_G_ < 0.001; *BF*_*Incl*_ = 0.240, and also no interaction, *F*(1,71) = 1.77; *p* = .188; η^2^_G_ = 0.001; *BF*_*Incl*_ = 0.307. In contrast, there was no changing-state effect, *F*(1,71) < 0.01; *p* = .938; η^2^_G_ < 0.001; *BF*_*Incl*_ = 0.131, no deviation effect, *F*(1,71) < 0.01; *p* = .960; η^2^_G_ < 0.001; *BF*_*Incl*_ = 0.124, and only a non-significant trend for (and Bayesian evidence against) an interaction, *F*(1,71) = 3.52; *p* = .065; η^2^_G_ = 0.003; *BF*_*Incl*_ = 0.086, on performance in the *mental arithmetic task* (note that again the pattern of results did not depend on inclusion or exclusion of the six participants who did not use a serial rehearsal strategy for serial recall).

### Discussion

Experiment 3 replicated the crucial interaction between the changing-state effect and the type of task with a disruptive effect demonstrated only in serial recall task, but not in a mental arithmetic task, which shared many of its properties with the serial recall task (e.g., stimulus presentation time, duration of exposure to sound). Interestingly, it was found that the majority of participants (91.4%) reported to either have silently repeated or grouped the digits during the serial recall task (a strategy that clearly cannot be applied to the mental arithmetic task), and the changing-state effect on serial recall was even more pronounced in these individuals (e.g., stronger Bayesian evidence for the interaction). The results thus indicate that changing-state sound is likely to have interfered specifically with the processing of serial-order (e.g., subvocal rehearsal), but they did not interfere with other mental operations involving digits (i.e., quick additions and subtractions).

Interestingly, there was again no evidence of a deviation effect in either task. In particular, the presence of a voice deviant in task-irrelevant steady-state and changing-state sound sequences did not disrupt performance in the serial recall task or the mental arithmetic task. Together with the absence of a deviation effect in the previous two experiments of this study, this finding suggests that the deviation effect on performance in a serial digit recall task may be a much less robust phenomenon than assumed (Bell, Mieth, et al., 2019; see also Kattner & Bryce, 2021). Although many procedural properties of Experiment 3 were identical to several previous studies (e.g., the presentation rate and the position of the deviant; Bell, Mieth, et al., 2019; Hughes et al., 2005; Marois et al., 2019), it is possible that distraction may rely on subtle procedural aspects that still differed between the present as well as the previous studies. For instance, a burst of pink noise in a sequence of spoken letters (Marois et al., 2019) may capture more attention than a change in voice. Also, the deviation effect might be stronger if the irrelevant sequences contained semantic information (sequences of words; Bell, Mieth, et al., 2019) compared to sequences of spoken letters. It could be discussed also whether a manual response format (writing the digits into a booklet) together with a response deadline (Hughes et al., 2007) may have strengthened the disruptive effect of a deviant. Most importantly, the presentation of steady-state and changing-state sequences in different blocks (as in Hughes et al., 2007) may have helped the formation of a predictive model of the background sound, making it less likely that the deviant can be integrated (thus increasing attentional capture). Unfortunately, following up on these possible explanations of the absence of a deviation effect is beyond the scope of this article. However, it is important to note that the procedural parameters used in Experiment 3 very much resemble the methodology used in previous studies that reported a “robust” deviation effect (i.e., a voice shift at a particular position in the stream). Recent evidence from our lab suggests that a disruptive effect of an auditory deviant in the serial recall task and in particular its susceptibility to task-encoding load – which suggests attentional diversion – may be highly sensitive even to minor modifications of the properties of the deviant and the task (Kattner & Bryce, 2021), thus questioning the generalizability of this effect.

## General discussion

Three experiments – one in the laboratory and two web-based experiments – were conducted to investigate the disruptive effects of (a) changing-state sound and (b) auditory deviants on performance in two different cognitive tasks: serial recall of digits and mental arithmetic. In line with the assumption of task-specificity of the changing-state effect (Hughes, 2014; Hughes et al., 2005; Jones et al., 1996), randomly changing irrelevant sound was found to disrupt serial recall in all three experiments, but it did not affect performance in three types of presumably non-serial mental arithmetic tasks requiring either successive additions and subtractions or judgments of the correctness of math equations. These results clearly suggest that changing-state sound produces specific interference with the processing of serial order (Hughes, 2014; i.e., due to the automatic processing of irrelevant order information in changing-state sound; Jones et al., 1996) rather than a general diversion of central attention from the focal task. In line with previous findings, the results of Experiment 3 suggest that the majority of participants indeed engaged in a serial rehearsal strategy (silently repeating or grouping the items; Hughes & Marsh, 2020) when memorizing sequences of visually presented digits.

In contrast, rehearsal of order information alone clearly cannot be a successful strategy to perform mental additions and subtractions or to judge math equations. In fact, both types of mental arithmetic tasks demand very little short-term memory capacity (only storing a single number or an interim result of the left-hand side of the equation in short-term memory; note that a secondary task was added in Experiment 2 to increase working memory load), but they require either transformations or continuous updating of the information held in working memory. While previous studies observed auditory distraction in similar mental arithmetic tasks (typically speech; Banbury & Berry, 1998; Hadlington et al., 2006; Perham et al., 2013; Perham & Macpherson, 2012), the present results suggest that these cognitive processes are not susceptible to distraction by changing-state sound. Specifically, there was no indication of a changing-state effect on mental arithmetic performance in Experiments 1 and 3, and changing-state sound was found to be disruptive only in combination with an auditory deviant (and in a dual-task context) in Experiment 2. It is thus possible that the previously observed effects of auditory distraction in mental arithmetic tasks was due to attentional capture rather than interference with the specific processes required to perform the task. Specifically, the irrelevant speech may have contained information (e.g., an interesting radio feature; Banbury & Berry, 1998; or ascending/descending two-digit numbers similar to the result of the mental arithmetic problems; Perham et al., 2013) that diverted attention from the focal task more than the repeated letters that were presented in the three experiments of this study. In other words, the change of sounds (different spoken letters) in the present study may not have elicited an attentional capture effect strong enough to cause distraction in a mental arithmetic task, but it still produced interference with serial-order processing in the typical serial digit recall task.

While the dissociation of the changing-state effect between tasks was observed in all three experiments, there was very little evidence of a deviation effect in both serial recall and the mental arithmetic tasks. In particular, a deviant voice in the sequence of irrelevant spoken letters (changing-state) disrupted performance only in the mental arithmetic task of Experiment 2 (evaluating math equations), but it did not affect the successive additions and subtractions of Experiments 1 and 3, and it also did not affect serial recall in all three experiments.

It is unclear why the deviant produced distraction only on the evaluations of math equations in Experiment 2, but not on the successive additions and subtractions required in Experiments 1 and 3. It has been argued that the deviation effect may depend on cognitive control (with high task load reducing the deviation effect; Hughes et al., 2013), and it is certainly possible that the cognitive load imposed by successive addition and subtraction problems differs from the load of evaluating math equations (requiring multiplications and additions/subtractions beyond the tens boundary while memorizing word pairs). Several previous studies found that enhanced perceptual task-encoding load (visually degraded to-be-remembered items; Hughes et al., 2013), enhanced demands for visual attention (requiring a local focus on the constituents of Navon letters; Marsh et al., 2020), and enhanced cognitive load (the degree of inhibitory control required for the suppression of word reading in a Stroop recall task; Hughes & Marsh, 2019) reduced or eliminated the deviation effect, whereas the same manipulations did not affect the disruptions produced by changing-states sound. These findings indicate that attentional and cognitive resources are required to process auditory deviants, thus leading to performance decrements only in case of low task load, while distraction is attenuating with increased task load. However, in the present study the cognitive load of the addition and subtraction problems (in particular with slow presentation rate in Experiment 1) is assumed to be lower than that in Experiment 2. Therefore, it is rather unlikely that the absence of a deviation effect on mental arithmetic was due to enhanced task load. It may also be the case that the task load of the addition and subtraction problems in Experiments 1 and 3 (with eight or five digits, respectively) was lower than what was imposed in previous studies (consisting of 15 digits to be added or subtracted; Banbury & Berry, 1998). However, again the lower task load should have made a deviation effect more likely (compare Hughes et al., 2013; Hughes & Marsh, 2019). On the other hand, lower cognitive load of the mental arithmetic tasks in Experiments 1 and 3 might have enabled participants to resolve the conflict between perceptually processed auditory distracters and the target information of the focal task (Lavie, 2005), thus reducing distraction at a cognitive level (see Kattner & Bryce, 2021 for a similar finding). Interestingly, the voice deviant was found to disrupt mental arithmetic performance in Experiment 2 only when it occurred in a sequence of changing-state sound, whereas no deviation effect was observed with steady-state sound, suggesting that the two types of distraction may not be independent. Specifically, both the continuous interference with serial-order processing (of the digits in the math equation) and attentional capture at a particular point may be required to disrupt performance in a mental arithmetic task, whereas participants may have been able to compensate for either type of distraction alone (e.g., through cognitive control).

Clearly, the absence of a deviation effect on serial recall is at odds with several previous studies (Bell, Mieth, et al., 2019; Hughes et al., 2007; Marois et al., 2019), and this discrepancy might be due to certain procedural aspects that differed between the studies (e.g., the presentation of semantic information or an acoustically more distinct deviant such as a burst of noise; see above). It could be argued that the deviants presented in the present experiments did not capture enough attention to cause distraction in serial recall, due to the relatively low unexpectedness or self-relevance of a voice change (e.g., compared to one’s own name or a “taboo” word; Röer et al., 2013, 2017). Further, it may be possible that the deviation effect requires more exposure to steady-state sound before the first deviant is presented (e.g., pre-training with 16 steady-state trials; Bell, Mieth, et al., 2019; Röer, Bell, Marsh, & Buchner, 2015b). It is nevertheless unclear why a very similar type of voice deviant (without pre-training) produced distraction in a very similar serial recall task in other previous studies (e.g., Hughes et al., 2007). However, in contrast to the present three experiments, Hughes et al. (2007) presented steady-state and changing-state sound in separate blocks. This difference might be crucial since a blockwise presentation of either type of sound (without a deviant) may have facilitated the formation of a predictive model of the background sound. The degree to which a deviant capture attention is expected to depend on how well it can be integrated in the predictive model. Due to the intermixed presentation of steady-state and changing-state sounds in the present experiments, the predictive model may have been less constrained (i.e., still open to change), and the deviant voice could thus be integrated more easily than in a blockwise presentation of sounds. While a full investigation of the effects of these procedural differences between studies is beyond the scope of this article, the replicated absence of a deviation effect (a voice change in a spoken sequence of letters; as in Hughes et al., 2007) on performance in a typical serial recall task across three experiments is certainly an important finding, which casts doubt on the robustness and/or the task-independence of the deviation effect (see also Kattner & Bryce, 2021; for another series of experiments in which the deviation effect occured only under very specific conditions).

Together, these findings are not entirely consistent with the assumption of two functionally distinct forms of auditory distraction, arguing that the automatic perceptual organization of changing-state sound (as compared to steady-state sound) interferes with the specific motor-planning processes during serial rehearsal, whereas an auditory deviant diverts central attentional resources from the focal task (see the “duplex-mechanism account”; Hughes, 2014; Hughes et al., 2005; Hughes & Marsh, 2017). Although the changing-state effect was observed only in serial recall, but not in mental arithmetic, a deviation effect was absent in serial recall as well as in the addition and subtraction tasks of Experiments 1 and 3 (in contrast to the account). Moreover, an auditory deviant produced distraction from the evaluation of math equations only when it occurred embedded in a changing-state sequence of sounds (Experiment 2), suggesting that the two types of distraction may not be independent (also in contrast to the account).

There is an additional methodological aspect of the present study that is worth a discussion. While Experiment 1 was conducted in the laboratory, the lockdown of our laboratory due to the COVID-19 pandemic forced us to conduct the other two experiments online with participants using their own computer and audio equipment at home to run the task. Despite the lack of experimental control over the exact presentation of stimulus material, the two web-based experiments produced results that are highly consistent with Experiment 1 in terms of the changing-state effect. Specifically, Experiment 2 may be the first demonstration of a changing-state effect on serial recall using a web-based task environment for data collection (note that the disruptive effect of irrelevant sentential speech has been investigated with an online task even before the pandemic; Kreitewolf et al., 2019), suggesting that auditory distraction can be studied quite reliably using online experiments. The present results are thus highly promising with regard to the viability of using online experimentation and internet-based data collection to study some forms of auditory distraction (for a similar outcome, see also Elliott et al., 2022). On the other hand, the disruptive effect of a voice deviant on serial recall (Hughes et al., 2007) could not be replicated in the two online experiments of this study, even though these experiments were designed specifically to increase the change to observe it. Thus, there is certainly a possibility that attentional capture effects can be elicited more effectively in the lab than in a task that is conducted at home. For instance, in an online study, participants may be exposed to additional unknown and uncontrolled background sounds (e.g., road traffic, housemates, doorbell), which may have captured a certain degree of attention throughout the task. This may have led to habituation of the attentional orienting response to irrelevant sounds (Banbury & Berry, 1997; Sörqvist et al., 2012), thus reducing or eliminating additional experimental deviation effects such as the typical attentional capture by a change in voice. Admittedly, the fact that deviants did not cause significant disruption of serial recall in the laboratory Experiment 1 either seems to indicate that the online environment may not have been the only factor that prevented a deviation effect. On the other hand, in contrast to the online experiments, the Bayesian statistics revealed moderate evidence for a deviation effect in Experiment 1, indicating that the type of environment (online vs. laboratory) may have been an important moderating variable of this effect.

Taken together, the three experiments of the present study demonstrated that the disruptive effect of irrelevant changing-state sound was specific to short-term memory tasks requiring serial-order processing (e.g., inner rehearsal or grouping of digits), whereas performance in two types of mental arithmetic tasks (addition/subtraction and math equations) was not affected by the presence of changing-state sound. There was no evidence for a general deviation effect on both serial recall and mental additions/subtractions of digits, but an auditory deviant in changing-state sound seems to have impaired the evaluation of math equations (in Experiment 2), suggesting that the disruptive effects of changing-state sound and auditory deviants on cognitive performance may not be independent. The findings are consistent with the assumption of changing-state sound to be automatically processed for perceptual grouping of ordered auditory objects, thus producing specific interference with an ongoing seriation process (Jones et al., 1996). However, an auditory deviant in the task-irrelevant stream of background sound did not seem to divert sufficient central attention from the focal task (whether serial recall or addition/subtraction), to produce reliable disruption of performance. More research is required to determine whether the absence of the deviation effect was due to specific procedural aspects of the present study (e.g., the use of a web-based task environment) or the degree of cognitive control required to perform the task (e.g., due to low task load or task engagement; Bell et al., 2020; Hughes et al., 2013).

## Footnotes

1 Single deviants were presented at positions 8, 12, 13, 16, or 22, double deviants were presented at the positions 6+16 or 16+19, and triple deviants were presented at positions 8+16+18, 9+18+20, or 9+12+18 of the 24-consonant sequence, with each corresponding to the presentation of a different math equation within the trial.

2 Only two participants reported other persons speaking during the task, but it was unclear how long and at what point this occurred. The data were included in the analysis, but we note that the overall pattern of results did not depend on an inclusion of their data.

3 We also ran this analysis with the subsample of 66 participants who reported to have used serial rehearsal as a memorization strategy during the serial recall task. This analysis again revealed the crucial interaction between sound and task to be significant and slightly more likely, *F*(2,130) = 3.70; *p* = .027; η^2^_G_ = 0.004; *BF*_*Incl*_ = 3.00. In addition, there was also significant main effects of task, *F*(1,65) = 66.18; *p* < .001; η^2^_G_ = 0.220; *BF*_*Incl*_ > 10^8^, and sound, *F*(2,130) = 3.72; *p* = .027; η^2^_G_ = 0.004; *BF*_*Incl*_ = 1.34, indicating lower performance during changing-state speech (*M* = .738; *SE* = .016) than during steady-state (*M* = .763; *SE* = .016) and silence (*M* = .757; *SE* = .016) across both tasks in these participants. A planned contrasts analysis revealed a significant contrast between changing-state and silence (*p* = .045), as well as between changing-state and steady-state (*p* = .011), but not between steady-state and silence (*p* = .571).
